# Human polyomaviruses JCPyV and MCPyV in urothelial cell carcinoma: a single institution experience

**DOI:** 10.3389/fonc.2023.1251244

**Published:** 2023-12-13

**Authors:** Faisal Klufah, Ghalib Mobaraki, Shuai Shi, Tom Marcelissen, Raed A. Alharbi, Mousa Mobarki, Shaia Saleh R. Almalki, Joep van Roermund, Axel zur Hausen, Iryna Samarska

**Affiliations:** ^1^ Department of Pathology, GROW-School for Oncology and Reproduction, Maastricht University, Medical Centre+, Maastricht, Netherlands; ^2^ Department of Laboratory Medicine, Faculty of Applied Medical Sciences, Al-Baha University, Al-Baha, Saudi Arabia; ^3^ Department of Medical Laboratories Technology, Faculty of Applied Medical Sciences, Jazan University, Jazan, Saudi Arabia; ^4^ Department of Urology, Maastricht University, Medical Centre+, Maastricht, Netherlands; ^5^ Pathology Department, Faculty of Medicine, Jazan University, Jazan, Saudi Arabia

**Keywords:** JCPyV, bladder cancer, polyomavirus, tumorigenesis, decoy cells, HPyV6, HPyV7, MCPyV

## Abstract

**Objective:**

Urothelial cell carcinoma (UCC) is the most common type of urinary bladder. JCPyV and BKPyV have been detected in the urine and tissue of urothelial cell carcinomas (UCC) in immunocompetent patients. Here, we investigated the presence of several HPyVs in UCC samples using diverse molecular techniques to study the prevalence of HPyVs in UCC.

**Methods:**

A large single-institution database of urine cytology specimens (UCS; *n* = 22.867 UCS) has previously been searched for decoy cells (*n* = 30), suggesting polyomavirus infection. The available urine sediments and formalin-fixed paraffin-embedded (FFPE) tissue samples of UCC patients were tested for the presence of JCPyV-LTAg expression by immunohistochemistry (IHC) labeled with SV40-LTAg antibody (clone: PAb416) and subsequent PCR followed by sequencing. In addition, the presence of the oncogenic Merkel cell polyomavirus (MCPyV) and the presence of human polyomavirus 6 (HPyV6) and 7 (HPyV7) DNA were tested with DNA PCR or IHC.

**Results:**

Of the 30 patients harboring decoy cells, 14 were diagnosed with UCC of the urinary bladder (14/30; 46.6%) before presenting with decoy cells in the urine. The SV40-LTAg IHC was positive in all 14 UCC urine sediments and negative in the FFPE tissues. JCPyV-DNA was identified in all five available UCS and in three FFPE samples of UCC (three of 14; 21.4%). Two UCC cases were positive for MCPyV-DNA (two of 14; 14.3%), and one of them showed protein expression by IHC (one of 14; 7.1%). All specimens were HPyV6 and HPyV7 negative.

**Conclusion:**

Our findings show the presence of JCPyV in the urine and UCC of immunocompetent patients. Moreover, MCPyV was detected in two UCC cases. In total, five UCC cases showed the presence of either JCPyV or MCPyV. The evidence here supports the hypothesis that these viruses might sporadically be associated with UCC. Further studies are needed to confirm the relevance of JCPyV or MCPyV as a possible risk factor for UCC development.

## Introduction

Urothelial cell carcinoma (UCC) is the most common type of bladder cancer (BC) (1, 2). Several factors are associated with an increased risk of BC development, including but not limited to schistosomiasis infection, smoking, and genetic predisposition (3). To date, it is estimated that up to 15% of the world’s cancer case burden is attributed to viral infections (4, 5). JC polyomavirus (JCPyV) and BK polyomavirus (BKPyV) are small, unenveloped, circular, and double-stranded DNA viruses, and both were the first human polyomaviruses isolated from patients in 1971 (6, 7). Recently, the urotheliotropic BKPyV has been increasingly well-studied and has been discussed as a possible oncogenic virus in the development of UCC in immunocompromised populations (8). JCPyV (HPyV2) is a neurotropic virus with a genome that shares about 75% homology with BKPyV (9). It has been identified as the etiological agent in the development of progressive multifocal leukoencephalopathy (PML), most commonly in immunocompromised individuals (9).

JCPyV (HPyV2) is acquired during early childhood, has shown about 80% seropositivity in humans, and has been described as a possible oncogenic virus in immunocompromised patients (10–12). In addition, JCPyV is known to remain latent or persistent in tubular epithelial cells of the kidney and urothelial tissue after primary infection (13). JCPyV large tumor antigen (LTAg) is located in the early region and has shown the capability to bind specifically with the p53 protein and retinoblastoma (pRB) protein, as known for some of the human polyomaviruses (HPyVs) (14–16). The presence of JCPyV-DNA was previously reported in many different tumor tissues, such as cervical, colorectal, gastric, lung, breast, brain, and urothelial cancers (17–23). The current number of members of HPyVs detected in various types of cancers has recently risen. Among them, Merkel cell polyomavirus (MCPyV) is the most recently identified human DNA tumor virus, which is clonally integrated into the majority of Merkel cell carcinoma (MCC) (24–26). MCPyV (HPyV5) has also been detected in the fresh frozen tissues of bladder cancer (27). Additionally, Husseiny and colleagues reported low levels of MCPyV viral load in urine specimens from immunosuppressed patients who were prospectively enrolled in kidney transplants (28). However, the potential role of HPyVs in UCC tumorigenesis is not fully elucidated.

Urine cytology using Papanicolaou staining is well accepted to reliably detect virally infected epithelial cells in the voided urine also known as “decoy cells”. These cells are characterized by enlarged nuclei and intranuclear inclusion cellular changes, as noticed in UCC neoplasia (29). The presence of decoy cells in urine has been used mostly as a marker to predict BKPyV and JCPyV reactivation (30, 31). In addition, immunohistochemical (IHC) immunolabeled with mouse anti-SV40 large T antigen monoclonal antibody, which is known to cross-react with both JCPyV and BKPyV LTAg, is used as an alternative marker to detect the reactivation of both respected viruses in urothelial cells in voided urine (10, 32, 33).

Recently, we studied the association between the BKPyV infection and UCC in patients with urine cytology positive for decoy cells (30). However, in our patient cohort, both primary and recurrent UCC tissues tested negative for BKPyV by PCR and IHC (30). Recent studies have investigated the role of JCPyV (HPyV2) in UCC tumorigenesis (34, 35). The aims of this study were to evaluate the presence of JCPyV, HPyV6, HPyV7, and MCPyV (HPyV5) in the UCC samples and in the voided urine of the patients diagnosed with UCC and with decoy cells in urine cytology.

## Materials and methods

### Patients and sample collection

**Figure 1:**
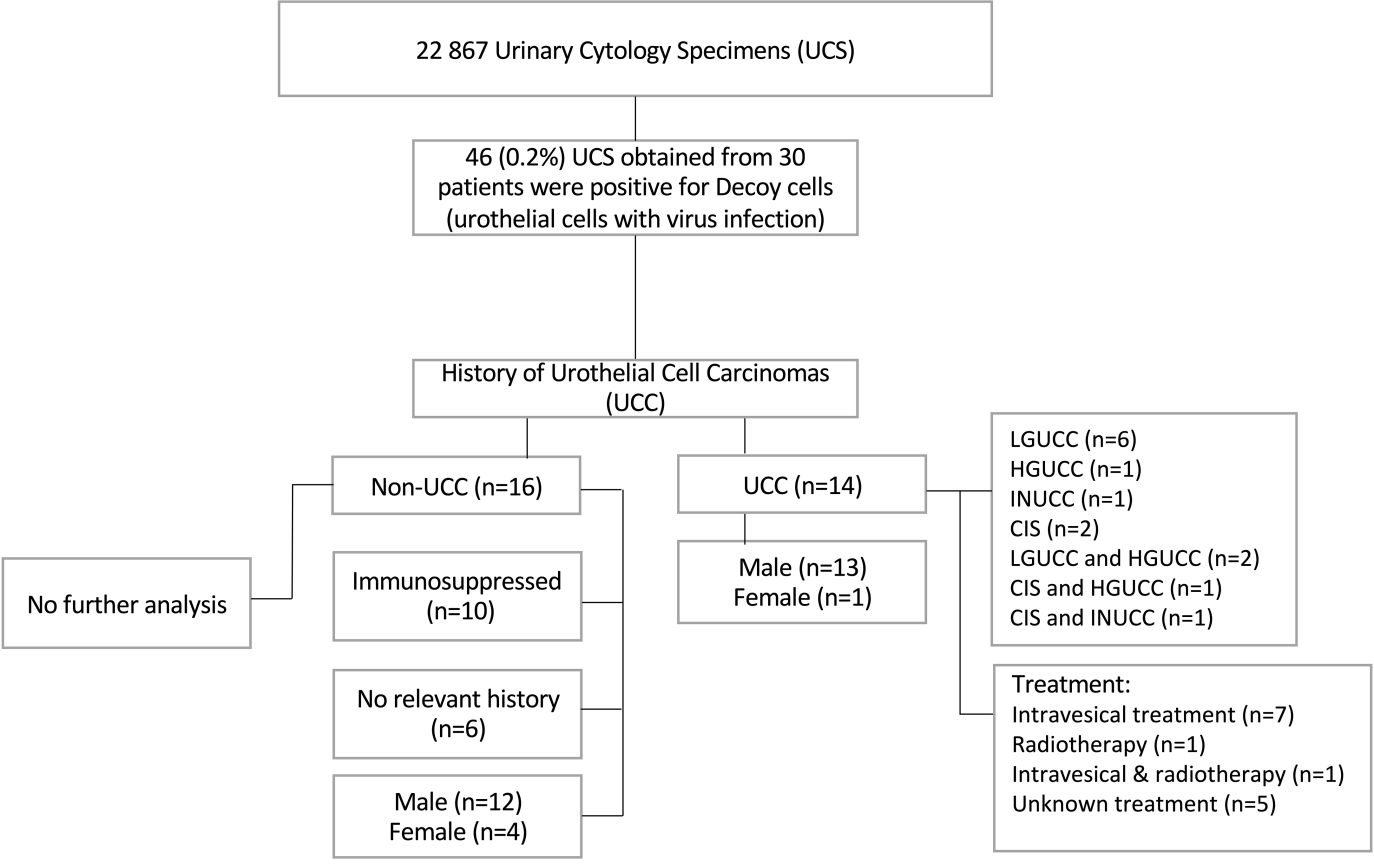
Flow chart summarizing the cohort clinicopathological data and urine cytology results. LGUCC, low-grade noninvasive urothelial cell carcinoma; HGUCC, high-grade noninvasive urothelial cell carcinoma; INUCC, invasive urothelial cell carcinoma; CIS, carcinoma in situ.

**Figure 2:**
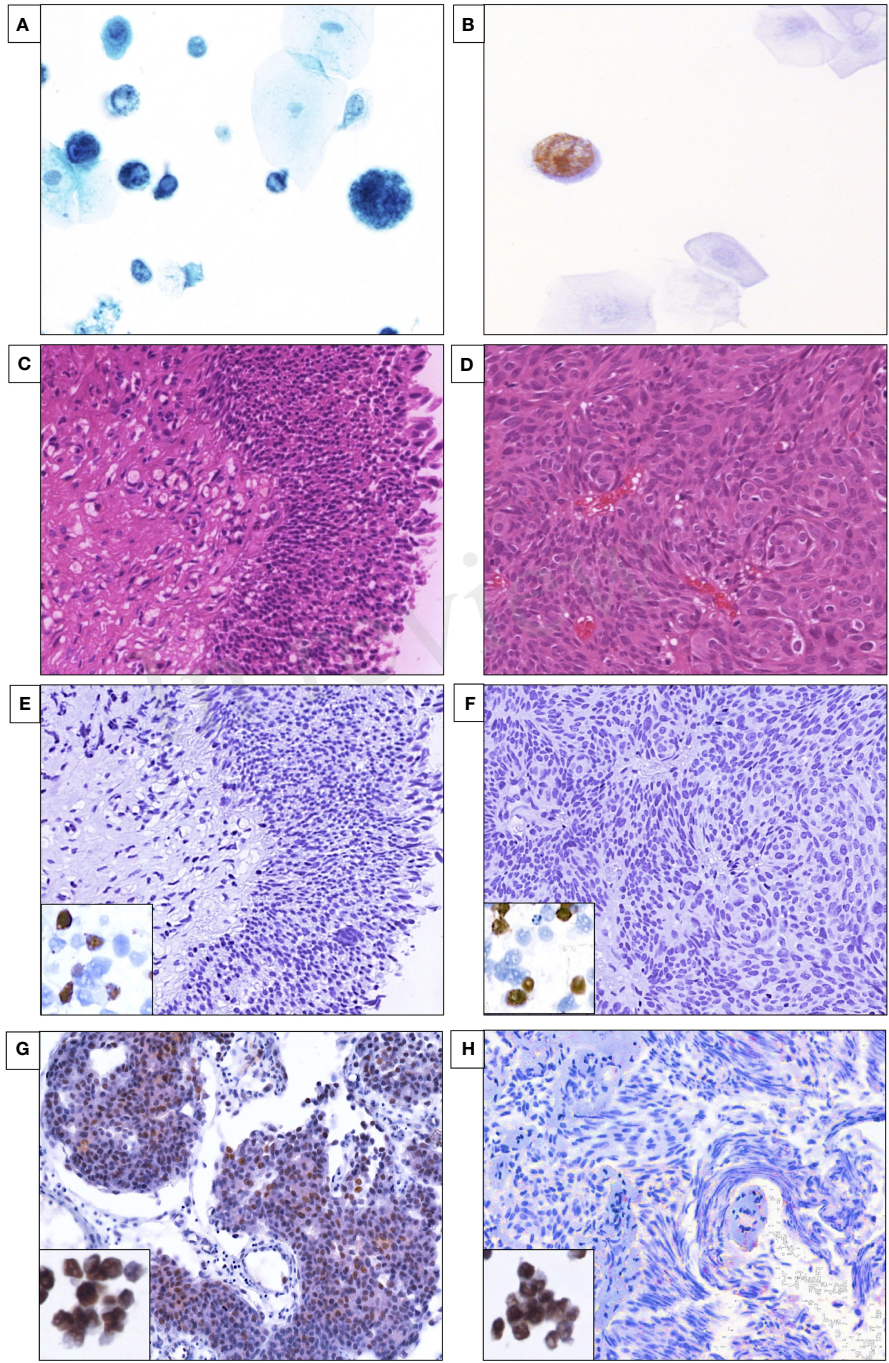
**(A)** Represents the decoy cells in urine cytology stained with Papanicolaou staining. **(B)** SV40-LTAg immunohistochemistry for UCC urine sediments. **(C)** H&E of low-grade noninvasive urothelial cell carcinoma (LGUCC). **(D)** H&E of high-grade noninvasive urothelial cell carcinoma (HGUCC). **(E)** SV40-LTAg immunohistochemistry for LGUCC FFPE tissues (left corner box is the positive control). **(F)** SV40-LTAg immunohistochemistry for HGUCC FFPE tissues (left corner box is the positive control). **(G)** Positive IHC for MCPyV, specific nuclear expression (brown) of MCPyV (CM2B4 antibody) in the nuclei of UCC epithelial (left corner box is the positive MKL1 control). **(H)** Negative IHC for MCPyV (left corner box is the positive MKL1 control).

Thirty urine cytology specimens (UCS) containing decoy cells were previously identified from a retrospective cohort study, including a total of 22,867 voided urine cytology specimens retrieved from the files of the Department of Pathology, MUMC+, The Netherlands, between January 2004 and December 2019, as reported previously (**Figures 1**, **2A**) (30). Clinicopathologic data were collected from the medical record, as described previously (**Figure 1**) (30). The SV40-LTAg IHC and bladder formalin-fixed paraffin-embedded (FFPE) specimens were collected from the archive of the Department of Pathology, MUMC+. All the slides were reviewed by two pathologists (IVS and AzH). The study was approved by the Medical Ethics Review Committee of the Maastricht University Medical Centre+, The Netherlands (2019-0977). All specimens were collected and studied in accordance with the protocol of the Dutch Code of Conduct for Observational Research with Personal Data (2004) and Tissue (36).

### Cytology, histology, and immunohistochemistry

Two slides were prepared for each urine sediment sample using the previously published Cytospin protocol (37). The first slide was stained with Papanicolaou stain, and the other slide was used for immunoperoxidase IHC using SV40-LTAg antibody (clone: PAb416, dilution 1:500, Calbiochem Inc, San Diego, CA, USA) with a Dako’s autostainer according to the routine diagnostic pathology department protocol.

The urinary bladder FFPE sections were stained with routine H&E staining for light microscopy. Additionally, the UCC FFPE were immunolabeled with SV40-LTAg antibody (clone: PAb416, dilution 1:500, Calbiochem Inc, San Diego, CA, USA) as described previously (30). Furthermore, 10 UCC FFPE tissues were tested for the expression of MCPyV large tumor antigen (LTAg) protein using the CM2B4 monoclonal antibody (clone: CM2B4, dilution 1:50; Santa Cruz Biotechnology Inc, Santa Cruz, CA, USA), and the MCC cell line MKL-1, which is positive for MCPyV, was used as a positive control for the CM2B4 antibody. Briefly, 5-μm-thick FFPE sections were immunolabeled with a Dako’s autostainer Link 48 according to the EnVision FLEX Visualization manufacturer’s kit (K8008, Dako, Carpinteria, CA, USA), and protein expression in the nucleus was interpreted as a positive. Next, all the IHC slide photo results were obtained by digital scanning with the VENTANA iScan-HT slide scanner (Roche Diagnostics Inc, Tucson, AZ, USA).

### DNA extraction and specific HPyV-DNA PCR

DNA was extracted from the available five urine sediments obtained from UCC patients and 14 UCC FFPE tissue blocks according to the protocol of genomic DNA isolation using NucleoSpin^®^ Tissue (Macherey–Nagel GmbH & Co, Düren, Germany). As previously described in our study, the DNA concentration, integrity, and quality were evaluated using a spectrophotometer (NanoDrop 2000, Thermo Scientific, Wilmington, DE, USA) and using multiplex primers [specimen control size (SCS)] as described previously (30, 38, 39).

JCPyV-DNA PCR was conducted by adding a total of 125 ng of DNA to the PCR master mix. Three sets of primers were used; the first set of primers was a previously published targeted conserved region of JCPyV and BKPyV LTAg using the following sequences: (FW: AAGTCTTTAGGGTCTTCTAC) (RV: GTGCCAACCTATGGAACAGA) (40). Another previously published set of primers was also used to amplify 391 bp of JCPyV noncoding control region gene (NCCR) (FW: TTCCTCCCTATTCAGCACTT) (RV: AAAACAGCTCTGGCTCGCAA) NCCR gene (41). In addition, a third set of primers designed by our group (FW: GCTCATACCTAGGGAGCCAA) (RV: CTGCTTTCCACTTCCCCTTGT) to target the JCPyV-NCCR gene were used to amplify 132 bp. The two sets of primers targeting JCPyV-NCCR were employed in order to distinguish between JCPyV strains, either archetype or Mad strains. To validate the PCR analysis, a known JCPyV-positive specimen was used as a positive control. In addition, DNA PCR targeting HpyV6, HpyV7, and MCPyV were performed using protocols as recently described (42, 43). DNA-PCR were used to amplify the small tumor antigen (sTAg) of HPyV6 and HPyV7, targeting the common region between sTAg and LTAg of MCPyV. HPyV6 and HPyV7 plasmids with inserted histidine tag and the MCPyV-positive MCC cell line (MKL-1) were used as positive controls. Bioperformance-certified water was used as a nontemplate negative control. In positive cases, all the amplified PCR products were purified, subsequently submitted for sequencing, and analyzed with the consensus sequences of the National Center for Biotechnology Information (NCBI).

## Results

### SV40-LTAg and MCPyV-LTAg IHC in FFPE tissues of UCC

No viral histomorphological cytopathic changes were seen in the UCC specimens of all 14 patients (**Figures 2C, D**). We previously assessed the expression of SV40-LTAg in all 14 UCC FFPE specimens and revealed no expression of SV40-LTAg in both invasive and *in situ* UCC samples (**Table 1**; **Figures 2E, F**) (30). UCC samples were assessed for MCPyV-LTAg by IHC, and one UCC sample tested positive (one of 10; 10%; **Table 1**; **Figure 2G**). The expression of MCPyV-LTAg revealed weak to moderate nuclear immunostaining in less than 60% of the tumor cells, and no cytoplasmic expression was observed in the neoplastic urothelium (**Table 1**; **Figures 2G, H**).

**Table 1:** Clinicopathological data, urine cytology, SV40 IHC, FFPE IHC, and JCPyV-DNA PCR results.

Specimen ID	G	Age	Diagnosis	Treatment	UCC FFPE tissues								Urine specimens							
					JCPyV-LTAg PCR	JCPyV-NCCR PCR (132 bp)	JCPyV-NCCR PCR (391 bp)	MCPyV-M1/M2 PCR	MCPyV-LATg IHC	SV40-LTAg IHC	HPyV6-sTAg PCR	HPyV7-sTAg PCR	Decoy cells	SV40-LTAg IHC	JCPyV-LTAg PCR	JCPyV-NCCR PCR (132 bp)	JCPyV-NCCR PCR (391 bp)	HPyV6-sTAg	HPyV7-sTAg	MCPyV-M1/M2 PCR
**1**	M	83	LGUCC	Mitomycin	+	+	−	−	−	−	−	−	+	+	n.a.	n.a.	n.a.	n.a.	n.a.	n.a.
**2**	M	71	LGUCC	Unknown	+	+	−	−	−	−	−	−	+	+	n.a.	n.a.	n.a.	n.a.	n.a.	n.a.
**3**	M	76	LGUCC	Unknown	+	+	−	−	−	−	−	−	+	+	n.a.	n.a.	n.a.	n.a.	n.a.	n.a.
**4**	M	90	INUCC	Unknown	−	−	−	+	+	−	−	−	+	+	n.a.	n.a.	n.a.	n.a.	n.a.	n.a.
**5**	M	59	LGUCC	Mitomycin	−	−	−	+	n.a.	−	−	−	+	+	+	+	+	−	−	−
**6**	M	70	CIS	BCG	−	−	−	−	−	−	−	−	+	+	+	+	+	−	−	−
**7**	M	72	LGUCC, HGUCC	BCG	−	−	−	−	n.a.	−	−	−	+	+	+	+	+	−	−	−
**8**	F	80	CIS and HGUCC	BCG	−	−	−	−	n.a.	−	−	−	+	+	+	+	+	−	−	−
**9**	M	78	LGUCC	Unknown	−	−	−	−	n.a.	−	−	−	+	+	+	+	+	−	−	−
**10**	M	89	LGUCC, HGUCC	BCG/mitomycin	−	−	−	−	−	−	−	−	+	+	n.a.	n.a.	n.a.	n.a.	n.a.	n.a.
**11**	M	55	CIS, INUCC	BCG/radiotherapy	−	−	−	−	−	−	−	−	+	+	n.a.	n.a.	n.a.	n.a.	n.a.	n.a.
**12**	M	74	CIS	BCG	−	−	−	−	−	−	−	−	+	+	n.a.	n.a.	n.a.	n.a.	n.a.	n.a.
**13**	M	66	HGUCC	Radiotherapy	−	−	−	−	−	−	−	−	+	+	n.a.	n.a.	n.a.	n.a.	n.a.	n.a.
**14**	M	55	LGUCC	Unknown	−	−	−	−	−	−	−	−	+	+	n.a.	n.a.	n.a.	n.a.	n.a.	n.a.

G, gender; LGUCC, low-grade noninvasive urothelial cell carcinoma; HGUCC, high-grade noninvasive urothelial cell carcinoma; INUCC, invasive urothelial cell carcinoma; CIS, carcinoma *in situ*; BCG, Bacillus Calmette–Guerin; +, positive; −, negative; FFPE, formalin-fixed paraffin-embedded tissues; UCC, urothelial cell carcinomas; PCR, polymerase chain reaction; LTAg, large tumor antigen; sTAg, small tumor antigen; NCCR, noncoding control region gene; n.a., not applicable; IHC, immunohistochemistry; HPyV6, human polyomavirus 6; HPyV7, human polyomavirus 7; MCPyV, Merkel cell polyomavirus; M1/M2, the common region between sTAg and LTAg.

### JCPyV-, HPyV6-, HPyV7-, and MCPyV-specific PCRs in FFPE tissues and urine of UCC

**Figure 3:**
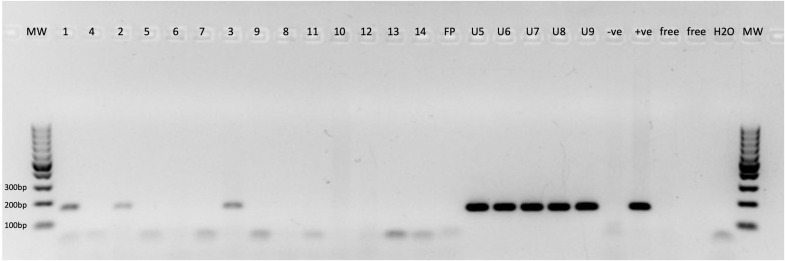
Specific JCPyV-DNA-PCR. 1–14, JCPyV-DNA PCR for urothelial cell carcinoma (UCC) FFPE tissue specimens; FP, free paraffin as negative control; U10–U14, JCPyV-DNA PCR for urine sediments. −ve, clinical specimen negative for JCPyV; +ve, clinical specimen positive for JCPyV; Free, empty slot (no master mix added to the gel); H_2_O, water nontemplate; MW, molecular weight marker.

JCPyV- (LTAg and NCCR) DNA-PCR was conducted on all 14 UCC FFPE specimens, and the results revealed that three samples (three of 14; 21.4%) tested positive using both JCPyV-LTAg and JCPyV-NCCR primer sets (primary and recurrent UCC) (**Table 1**; **Figure 3**). Sequencing analyses of the PCR amplicons confirmed that all PCR products were 96% to 98% identical to JCPyV. Interestingly, all three UCC specimens, positive for JCPyV-LTAg and JCPyV-NCCR PCR, were low-grade noninvasive urothelial cell carcinoma (LGUCC) (**Table 1**). The other five patients with UCC, initially negative for JCPyV LTAg and NCCR, showed positivity for this virus in their urine sediments (**Table 1**). Of note and important to remember, urine cytology was taken in a follow-up period after the initial UCC resection, and four of these patients received an intravesical treatment (BCG or mitomycin). The JCPyV-LTAg and JCPyV-NCCR were amplified in all five specimens and confirmed by sequencing with homology that ranges from 97% to 99% identical to JCPyV (**Table 1**; **Figure 3**). Furthermore, using JCPyV-NCCR PCR revealed that the urine and tissue specimens were positive for the JCPyV harbor archetype strain. Additionally, HPyV6-, HPyV7-, and MCPyV-specific PCR were conducted on all 14 UCC FFPE samples and five urine sediments. Two UCC samples (two of 14; 14.3%) were positive for MCPyV PCR targeting the common region of LTAg and sTAg. One patient presented with MCPyV in the UCC FFPE specimen and JCPyV-LTAg in the urine sediment (ID5, **Table 1**). Neither HPyV6- nor HPyV7-DNA could be amplified by PCR (**Table 1**).

## Discussion

We recently reported that we did not find an association between BKPyV detection in the urine sediments of patients with prior resected urothelial cell carcinomas. BKPyV reactivation was not restricted to immunosuppression and was possibly related to intravesical BCG or mitomycin treatment in immunocompetent patients (30). JCPyV is closely related to BKPyV, and the large- and small-tumor antigens of both viruses were found to be capable of transforming cells *in vitro* (44–47). In contrast to the relationship of BKPyV with UCC, limited information is available regarding the role of JCPyV in UCC tumorigenesis, especially in immunocompetent cases (34, 35).

We analyzed the presence of JCPyV on the protein level in combination with DNA-PCR in FFPE tissues and voided urine sediments obtained from UCC immunocompetent patients. Previously, we demonstrated that the detection of decoy cells was infrequent, present in only 0.2% of a large urine specimen cohort from our institution (30). The detection of decoy cells in urine cytology is used in a diagnostic setting for identifying possible polyomavirus infections (48). However, the decoy cells were identified in the urine cytology of all 14 UCC cases, which suggests polyomavirus reactivation (30). Indeed, SV40-LTAg expression was detectable in the urine sediments, which confirmed polyomavirus reactivation in all 14 UCC patients (**Figure 2B**). Although the SV40-LTAg monoclonal antibody is commonly used in diagnostic pathology, it is known to cross-react with the closely related human polyomaviruses BKPyV and JCPyV (49). We confirmed the presence of JCPyV-LTAg by PCR among the five available urine sediments obtained from five UCC patients. Also, we previously reported the detection of BKPyV-LTAg by PCR in all of the five urine sediments (30). Thus, it can be speculated that the tubular epithelial cells are coinfected with both JCPyV and BKPyV, and both may contribute to the presence of the decoy cells.

Here, we performed JCPyV-LTAg and JCPyV-NCCR PCR in UCC FFPE samples obtained from patients who were diagnosed with decoy cells in their urine. It is important to mention that the patients were diagnosed with UCC, and urine cytology was collected during the postoperative follow-up. The main finding in our data is that 21.4% of UCC FFPE tissues tested positive using JCPyV LTAg and NCCR PCR, as confirmed by sequencing. Furthermore, the JCPyV-NCCR positive tissues and urine specimens were identical to the archetype strain, as confirmed by the presence of the 64 and 23 bp sequences that are absent in the Mad strains, which have been associated with human brain tumors. However, there is a study that reported the detection of archetype strain sequences in two cases of oligodendroglioma (50). The detection of JCPyV-DNA was previously reported in many different tumors, such as cervical, colorectal, gastric, lung, breast, brain, and urothelial cancers (17–23). Infection with JCPyV has been suggested to be associated with bladder carcinoma, which still remains a controversial hypothesis (34, 35). Results from a study that screened for JCPyV among urothelial bladder cancer patients in the UK concluded that 0.9% of bladder tumors were positive for JCPyV (34). In contrast, Fioriti et al. suggested that JCPyV might play a role in bladder cancer and reported the detection of JCPyV-DNA in 19% of bladder urothelial carcinoma (35).

JCPyV is known to remain latent in the kidney after infection and reactivate in immunosuppressed patients (11). However, in our study, JCPyV-PCR positivity in urine sediments was seen in immunocompetent UCC patients; most of the patients were known to have received either mitomycin or intravesical BCG treatment. Thus, our results suggest that the reactivation of latent JCPyV could be related to the locally administered UCC intravesical treatment since the patients were diagnosed with UCC of the urinary bladder before presenting with decoy cells in the urine cytology.

MCPyV is an oncogenic virus discovered in 2008 and has been linked to the pathogenesis of the majority of MCC. MCC are rare, highly aggressive neuroendocrine nonmelanoma skin cancers (24–26). In contrast, there is yet no evidence established regarding the role of MCPyV in bladder carcinoma. One study reported the presence of MCPyV-DNA in voided urine specimens of transitional cell carcinomas of the bladder and revealed 2.7% positivity by PCR (51). Also, Loyo et al. reported the detection of MCPyV-DNA in 75% of bladder cancer tissues with a low viral load (< 0.001 copies per genome) (27). Another study revealed that MCPyV was detected in 30% of the urine specimens from prospectively enrolled immunosuppressed kidney transplant patients by using qPCR with a relatively low viral load (10 to 3.7 × 10^2^ genome copies/mL) (28). In our study, MCPyV was not detectable in the urine sediments of UCC, while MCPyV-DNA was positive in two 14.3% UCC FFPE tissues, and specific nuclear MCPyV-LTAg expression was seen in the neoplastic cells of one UCC tissue. Indeed, it is very interesting that MCPyV immunoreactivity was found to be abundantly present in UCC tumor cells. However, a direct contributing role of MCPyV to UCC carcinogenesis seems unlikely, at least based on our findings unlikely (24–26). The observed pattern of MCPyV protein expression in UCC tissues possibly might imply an indirect role for MCPyV in the development of UCC by, e.g., inflammation as it is known for hepatotropic viruses (e.g., hepatitis B and C viruses) (52). However, further investigations are needed to identify any oncogenic mutations or clonal integration of MCPyV LTAg or sTAg into the genome of UCC.

Noteworthy, the mouse anti-SV40-LTAg monoclonal antibody (clone PAb416) is commonly used in clinical pathology settings and is well known to cross-react with both JCPyV and BKPyV LTAg (53). However, Toptan et al. reported that anti-SV40-LTAg (PAb416) is not restricted to JCPyV and BKPyV. Of note, PAb416 also detects LTAg proteins of KIV, WUV, HPyV6, HPyV7, TSV, HPyV10, and HPyV11 (54). HPyV6 and HPyV7 share approximately 68% genome sequence identity and reveal high seropositivity in healthy populations (55–57). Since anti-SV40-LTAg showed the ability to detect both HPyV6 and HPyV7, we have tested all 14 UCC tissues and the five urine sediments for the presence of HPyV6- and HPyV7-DNA. Our results showed that all UCC specimens were negative for both HPyV6 and HPyV7, and to our knowledge, this is the first study to test UCC specimens for HPyV6 and HPyV7.

Some limitations of this study need to be mentioned. First, the size of the study cohort is relatively small, which partially reflects the rarity of UCC and the number of UCC patients admitted to our hospital. Second, obtaining healthy control tissues is practically impossible. In addition, parallel screening for HPyV-DNA in blood specimens would have been an interesting add-on to our study; however, we were unable to carry out these experiments due to the unavailability of blood.

To the best of our knowledge, this is the first study to investigate the presence of several HPyVs in UCC samples using diverse molecular techniques and the possible contribution of HPyVs in immunocompetent UCC etiopathogenesis. In total, five UCC cases showed the presence of either JCPyV or MCPyV. Both HPyV6 and HPyV7 were not detected. Further studies are warranted to confirm the relevance of JCPyV or MCPyV as a risk factor for developing UCC and to identify any oncogenic mutations or clonal integration into the genome of urothelial cell carcinoma.

## Conclusions

JCPyV-DNA is detected in the urine and urothelial cells, and MCPyV was detected in urothelial cell carcinoma. Since there is inadequate evidence of a role for JCPyV in carcinogenicity in UCC, these findings support the hypothesis that JCPyV infection could contribute to urothelial carcinoma tumorigenesis. Moving forward, it is important to define whether or not both JCPyV and MCPyV are involved in UCC tumorigenesis.

## Data availability statement

The original contributions presented in the study are included in the article/supplementary material. Further inquiries can be directed to the corresponding authors.

## Ethics statement

The study was approved by the Medical Ethics Review Committee of the Maastricht University Medical Centre+, The Netherlands (2019-0977). All specimens were collected and studied in accordance with the protocol of the Dutch Code of Conduct for Observational Research with Personal Data (2004) and Tissue. The studies were conducted in accordance with the local legislation and institutional requirements. The human samples used in this study were acquired from a by- product of routine care or industry. Written informed consent for participation was not required from the participants or the participants’ legal guardians/next of kin in accordance with the national legislation and institutional requirements.

## Author contributions

All authors have contributed substantially to conceptualizing the research design planning and continued supervision of the work (FK, GM, AH, IS), aacquisition of data (FK, GM, SS, TM, RA, MM, JR), performed and processed the experimental data and analysis (FK GM SS RA, SA), analysis and interpretation of data (FK, GM, TM, MM, SA, AH, IS), drafting the manuscript (FK, GM, AH, IS), and revising it critically (JR, AH, IS). All authors contributed to the article and approved the submitted version.
